# Pediatric densitometry: is the Z score adjustment necessary in all cases?

**DOI:** 10.3389/fendo.2025.1587382

**Published:** 2025-04-09

**Authors:** Berta Magallares, Jorge Malouf, Helena Codes-Méndez, Hye Sang Park, Jocelyn Betancourt, Gloria Fraga, Estefanía Quesada-Masachs, Mireia López-Corbeto, Montserrat Torrent, Ana Marín, Silvia Herrera, Ignasi Gich, Susana Boronat, Jordi Casademont, Hector Corominas, Dacia Cerdá

**Affiliations:** ^1^ Department of Rheumatology, Hospital de la Santa Creu i Sant Pau, Barcelona, Spain; ^2^ Department of Rheumatology, Universitari Dexeus-Grupo Quirón Salud Hospital, Barcelona, Spain; ^3^ Institut de Recerca Sant Pau (IR SANT PAU), Sant Quintí 77-79, Barcelona, Spain; ^4^ Medicine Faculty, Universitat Autònoma de Barcelona (UAB), Cerdanyola del Vallès, Spain; ^5^ Department of Mineral Metabolism Unit - Internal Medicine, Hospital de la Santa Creu i Sant Pau, Barcelona, Spain; ^6^ Department of Pediatrics, Hospital de la Santa Creu i Sant Pau, Barcelona, Spain; ^7^ Department of Pediatric Rheumatology, Vall d’Hebrón Barcelona Hospital, Barcelona, Spain; ^8^ Centro de Investigación Biomédica en Red de Enfermedades Raras (CIBERER), Madrid, Spain; ^9^ Department of Clinical Epidemiology and Public Health, Hospital de la Santa Creu i Sant Pau, Barcelona, Spain; ^10^ Department of Internal Medicine, Hospital de la Santa Creu i Sant Pau, Barcelona, Spain; ^11^ Department of Rheumatology, Hospital Sant Joan Despí Moisès Broggi, Barcelona, Spain

**Keywords:** pediatric densitometry, height-for-age z-score, bone mineral density, low bone mineral mass, DXA (dual-energy x-ray absorptiometry)

## Abstract

**Background:**

The International Society for Clinical Densitometry recommends adjusting the bone mineral density (BMD) Z-score in children with short stature or growth delay. However, it is not clear whether height-for-age Z-score (HAZ) adjustment is required in all children. The aim of this study was to determine whether HAZ adjustment is necessary by examining variability in unadjusted and adjusted Z-scores for the main regions of interest in a large pediatric cohort.

**Methods:**

We evaluated 103 patients ≤ 20 years of age who underwent lumbar spine and whole-body dual-energy x-ray absorptiometry (DXA) at our tertiary care hospital from 2016 to 2018. The formula proposed by Zemel was used to calculate the HAZ.

**Results:**

A total of 103 participants were included (54 females; 52.4%). The mean age was 9.8 years. Height percentiles were ≤ 3 or ≥ 97 in seven (6.8%) and five (4.9%) patients. Diagnostic criteria for low bone mineral density (LBMD; BMD Z-score ≤ −2) were met in 8 lumbar spine scans and 10 whole-body scans. After HAZ adjustment, the prevalence of LBMD decreased from 8.2% (n=8) to 6.4% (n=6) in the lumbar spine scans and from 10.5% (n=10) to 7.2% (n=8) in the whole-body scans. Agreement between the adjusted and non-adjusted HAZ data was 0.498 for the lumbar spine and 0.557 for the whole body. The diagnostic discrepancy rate for LBMD diagnosis was 7%. After HAZ adjustment, 5% patients no longer met LBMD criteria while conversely 2% met LBMD criteria only after adjustment.

**Conclusions:**

The high diagnostic discrepancy rate (7%) for LBMN in this unselected pediatric cohort underscores the value of performing HAZ adjustment of Z-scores to improve diagnostic accuracy. This divergence between adjusted and unadjusted Z-scores suggests that all pediatric patients, not only those with short stature or growth retardation, may benefit from densitometric size adjustment. This is especially true in individuals whose stature is at the upper end of the range, where size may obscure a diagnosis of LBMD.

## 1 Introduction

Several imaging techniques are currently available to monitor bone disease in the pediatric population (1). The gold standard for measuring bone mineral content (BMC) and bone mineral density (BMD) is dual-energy X-ray absorptiometry (DXA) (2, 3). DXA has several advantages, including low radiation doses (4–27 μSv), a short scanning time, widespread availability, high reproducibility, and an extensive body of pediatric reference data (4). Moreover, DXA plays a key role in assessing pediatric bone health because the definition of low bone mineral density (LBMD) is based on densitometric criteria (3, 5).

Interpreting DXA results in children can be challenging due to the dynamic nature of the growing skeleton. Unlike adults, children’s bones grow and change their tridimensional shape over time, and growth is highly variable depending on the individual and the developmental stage (5). DXA is a two-dimensional (2D) imaging technique that relies on differential X-ray absorption to distinguish between tissues of varying densities. Unfortunately, it cannot measure the depth of bones, the third dimension. As a result, BMD is calculated as a 2D projection of a 3D structure, expressed in g/cm2 (5, 6). Because BMD is not a volumetric measure, it is influenced by the size of the bone being assessed. Consequently, smaller bones may appear to have a lower BMD than larger bones, even if their actual volumetric density is identical (5), which means that DXA-derived areal BMD tends to underestimate true volumetric BMD (g/cm³) in children with short stature while overestimating BMD in taller children (7, 8). For this reason, in 2019, the International Society for Clinical Densitometry (ISCD) officially recommended adjusting BMD Z-scores for bone size in children with growth impairment (3).

Several different approaches have been developed to adjust BMD Z-scores to more accurately determine BMD in children of all statures. Some of the more common techniques include adjustments based on height, weight, bone mineral apparent density, and the height-for-age Z-score (HAZ). While all of these methods have been shown to more accurately predict fracture risk in the pediatric population compared to unadjusted techniques (9), the HAZ-adjustment technique developed by Zemel et al. is considered to be the least biased method (2).

Beyond HAZ-based adjustments, other techniques have been explored to mitigate the impact of bone size on BMD measurements. These include adjustments based on bone age, pubertal stage, lean body mass, and vertebral body height. Some DXA systems also estimate bone volume using dimensional indices of the scanned region, enabling the calculation of volumetric BMD (vBMD) under simplified anatomical assumptions. For example, Kröger et al. proposed a method that considers the vertebrae as a cube or cylinder, using the formula: vBMD = aBMD × [4/(π × width)], where aBMD is areal BMD and width is the measured vertebral body width (10). In addition, new imaging technologies such as Radiofrequency Echographic Multi Spectrometry (REMS) are emerging as potential alternatives to DXA, with the advantage of assessing bone quality and fracture risk without being influenced by bone size (11). However, these technologies ae still undergoing validation and are not yet widely available in routine pediatric clinical practice.

In this context, the aim of this study was to compare standard BMD Z-scores to HAZ-adjusted Z-scores in a real-life cohort of pediatric patients. The study was carried out in the context of routine clinical practice in an unselected population. We sought to determine whether there were discrepancies in the diagnosis of LBMD based on HAZ adjusted and non-adjusted Z-scores for the main regions of interest.

## 2 Materials and methods

### 2.1 Study population

This was a cross-sectional, single-center study based on data from spine and whole-body DXA scans performed between 2016 and 2018. The study population included patients ≤ age 20 referred to the pediatric rheumatology outpatient clinic at our hospital for bone health assessment due to the presence of one or more of the following risk factors: malabsorption syndrome or food allergies; juvenile idiopathic arthritis; nephropathy; hematological disorders; systemic autoimmune or autoinflammatory disease; endocrinopathy; treatment with drugs that alter bone metabolism (e.g., glucocorticosteroids or immunosuppressants); lack of physical activity; or insufficient calcium intake.

The study was approved by the ethics committee at our hospital (IIBSP-FRA-2016-11). Informed consent was obtained from all patients and/or their legal guardians prior to recruitment.

### 2.2 Data collection and study variables

The following data were collected: date of birth; weight; height; and calculated height and weight percentiles. The presence of any of the aforementioned risk factors for developing low bone mass was recorded. Densitometric measurements included total body and subtotal body less head BMD; L1-L4 vertebrae BMD; and Z-scores for both total body and L1-L4 vertebrae. We did not include Z-scores for the total body less head (TBLH), as our densitometer lacks population-based normative data required for this calculation, a common issue in real-world clinical settings at many hospitals. HAZ adjustment for lumbar spine and total body Z-score values was applied in all cases using the formulas developed by Zemel et al. (8). Densitometric measurements were obtained with the Hologic Discovery densitometer scanner (Hologic, Inc., Bedford, MA, USA).

### 2.3 Statistical analysis

The IBM-SPSS software package (v. 26.0) was used to perform the statistical analyses. Quantitative variables are presented as means with standard deviation (SD) and categorical variables are presented as absolute frequencies with percentages. Distribution normality for the study variables was assessed using the Shapiro-Wilk test. Depending on the distribution, the differences in mean BMD according to Z-scores and HAZ scores were analyzed using the following tests, as appropriate: T-test, Mann-Whitney U test, Kruskal-Wallis test, or ANOVA for continuous variables; and Chi-square test or Fisher’s exact test for categorical variables. Pearson’s linear correlation coefficient or Spearman’s correlation coefficient were used to examine correlations between BMD values and HAZ-adjusted and unadjusted Z-scores. Analyses were conducted as two-tailed tests with a significance level set at 5% (α=0.05).

## 3 Results

### 3.1 Baseline characteristics and densitometric measurements

A total of 103 patients were included in the study. The mean age was 9.8 years. Height percentiles were ≤ 3 or ≥ 97 in seven (6.8%) and five (4.9%) patients. The baseline characteristics of the study population are shown in **Table 1**.

**Table 1:** Baseline characteristics.

Variable	n (%)
Sex, female	54 (52.4)
Age (years; range)	
Early childhood (2-3)	9 (8.7)
Childhood (4-9)	33 (32)
Adolescence (10-17)	55 (53.4)
Young adulthood (18-20)	6 (5.8)
Ethnicity	
Caucasian	82 (79.6)
Hispanic	10 10.7)
Arab-Berber	7 (6.8)
Other	3 (2.9)
Anthropometric characteristics	
Height ≤ 3^rd^ percentile	7 (6.8)
Height ≥ 97^th^ percentile	5 (4.9)
Weight ≤ 3^rd^ percentile	8 (8.7)
Weight ≥ 97^th^ percentile	9 (8.7)
Comorbid disease	99 (96.1)
Malabsorption/food allergies	47 (46.6)
Juvenile idiopathic arthritis	18 (17.5)
Nephropathy	18 17.5)
Hematological disorder	6 (6.8)
Systemic autoimmune disease	9 (7.8)
Autoinflammatory disease	3 (2.9)
Endocrinopathy	1 (1)

Densitometric data according to the region of interest are presented in **Tables 2**, **3**. **Table 2** shows BMD and BMC values for the lumbar spine with raw and HAZ Z-scores. **Table 3** provides BMD and BMC values for the whole body and TBLH with raw and HAZ Z-scores.

**Table 2:** Densitometric data for the lumbar spine.

	n	Mean	SD	Median	Range
BMC, g	102	29	15.89	25.52	6.20 – 90.40
BMD, g/cm^2^	102	0.64	0.18	0.61	0.36 – 1.06
Z-score	99	-0.46	1.08	-0.40	-3.20 – 1.80
HAZ-adjusted Z-score	95	-0.43	0.96	-0.41	-2.77 – 1.65

SD, standard deviation; BMC, bone mineral content; BMD, bone mineral density; HAZ, height-for-age Z-score.

**Table 3:** Densitometric data for the whole-body.

	n	Mean	SD	Median	Range
BMC, g	103	1172.2	520.6	1083.8	190.5 – 2326.4
BMC less head, g	98	861.9	443.8	775.7	244.4 – 1899.9
BMD, g/cm^2^	98	0.81	0.16	0.81	0.53 – 1.17
BMD less head, g/cm^2^	98	0.69	0.17	0.70	0.39 – 1.01
Z-score	96	-0.32	1.18	-0.20	-3.20 – 2.10
HAZ-adjusted Z-score	92	-0.40	1.02	-0.32	-3.41 – 1.77

SD, standard deviation; BMC, bone mineral content; BMD, bone mineral density; HAZ, height-for-age Z-score.

*Correlations between anthropometric measures and bone mineral density*

Both weight and height were significantly correlated with BMD at all three main locations (p<0.001 in all cases). Correlation coefficients for weight and height, respectively, were as follows: lumbar spine BMD: 0.855 and 0.824; whole body BMD: 0.889 and 0.899; and TBLH BMD: 0.908 and 0.935.

LBMD criteria (Z-score ≤2) were met by 8.2% (n=8) of the sample in the lumbar spine and 10.5% (n=10) in the whole-body. After HAZ adjustment, these values decreased to 6.4% (n=6) and 7.2% (n=8), respectively.

### 3.2 Comparison of unadjusted and HAZ-adjusted Z-scores

No significant differences (p=0.913) were observed in the mean unadjusted (-0.44 +/- 1.07) or HAZ-adjusted (-0.43 +/- 0.96) lumbar spine Z-scores. The correlation coefficient between the two scores was 0.78 (p<0.001), with a mean difference of 0.0075. However, the unadjusted Z-score was more variable than the HAZ-adjusted spine Z-score with a difference of 0.67.

**Figure 1:**
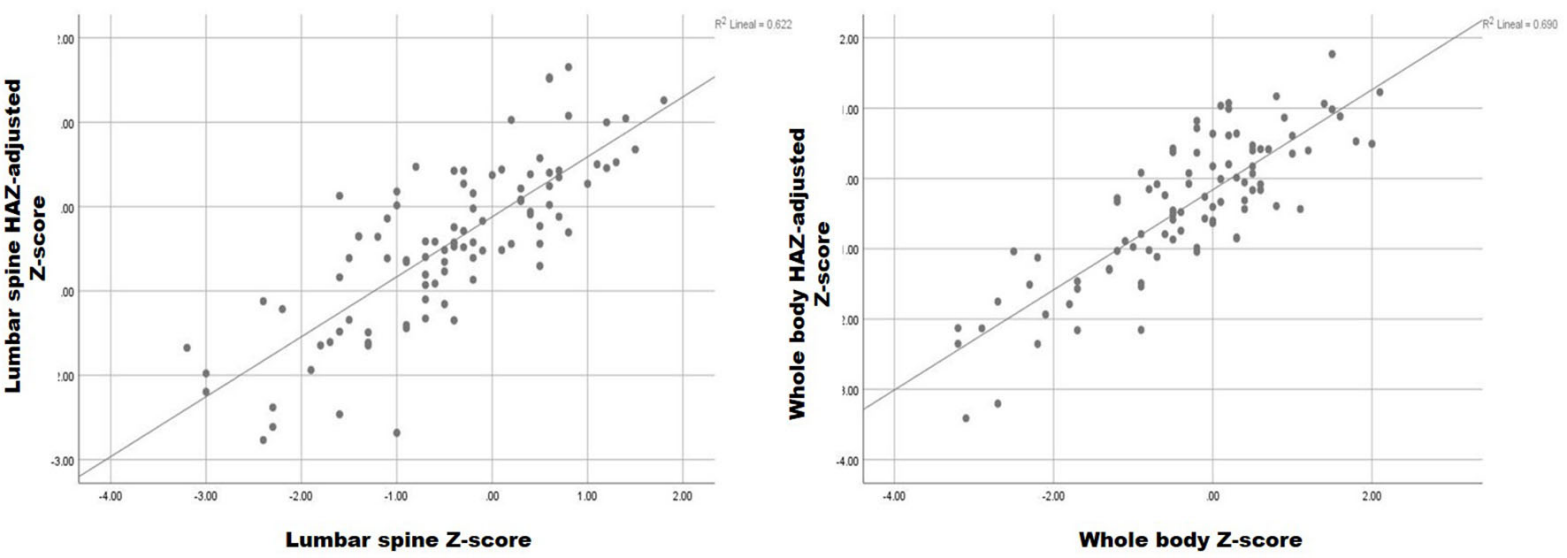
Relationship between HAZ-adjusted and unadjusted lumbar spine and whole-body Z-score.

The relationship between these variables is graphically illustrated in **Figure 1**. Despite the close correlation between the HAZ adjusted and unadjusted measures, there was a difference in the diagnosis of LBMD in 7% of the patients. More specifically, in 5% (n=5) of the patients, the unadjusted lumbar spine Z-score was ≤ -2 but the adjusted score was > -2. By contrast, in 2% (n=2) of patients, the HAZ-adjusted Z-score was ≤ -2 with an adjusted score > -2. At the LBMD threshold (≤ -2), the concordance index between the unadjusted and HAZ-adjusted lumbar spine Z-scores was 0.498.

No significant differences (p=0.367) were observed between the unadjusted (-0.34 ± 1.19), and HAZ-adjusted (-0.40 ± 1.02) whole body Z-scores, with a mean difference of 0.063 (-0.34 unadjusted vs -0.4 HAZ-adjusted Z-scores). The difference between the two measures in terms of SD was 0.66, indicating less dispersion in the HAZ-adjusted Z-score. The correlation between these variables was 0.82, as shown in **Figure 1**.

Two patients (2%) with a HAZ-adjusted whole-body Z-score ≤ -2 (LBMD threshold), had an unadjusted Z-score > -2. Similarly, 5 patients (5%) with an unadjusted Z-score ≤ -2, had a HAZ-adjusted Z-score > -2. The concordance between the unadjusted and the HAZ-adjusted whole-body Z-scores at the LBMD threshold was 0.557.

## 4 Discussion

The aim of this study was to determine whether size adjustment should be performed only in children with short stature (height < 3rd percentile), as currently recommended, or if it should always be adjusted when performing pediatric bone densitometry. This study was prompted by real-world clinical observations at our center, which led us to believe that the diagnostic accuracy of LBMD could be improved in other groups by performing HAZ adjustments, particularly in children with tall stature.

In this context, we determined the variability in densitometric Z-scores for the main ROIs, with and without HAZ Z-score adjustment, in a cohort of children who were not pre-selected based on height. We adjusted all Z scores, regardless of the individual’s height or weight percentile. We found no significant differences in the mean adjusted and unadjusted Z-scores for the main ROIs (i.e., spine and whole body). However, the HAZ-adjusted Z-scores showed less dispersion (based on SD values), indicating reduced variability.

At the LBMD threshold (Z-score ≤ -2), the discordance rate between HAZ-adjusted and unadjusted measurements was 7% in the two ROIs (lumbar spine and whole body). After HAZ adjustment, 5% of patients who had met diagnostic criteria for LBMD (based on unadjusted values) no longer met those criteria and 2% of patients whose Z-scores were considered normal actually met LBMD criteria.

After HAZ adjustment, 5% of patients who initially met diagnostic criteria for LBMD no longer did, likely corresponding to those on the lower end of the height percentile. Conversely, 2% of patients who now met LBMD criteria post-adjustment were likely on the higher end of the height percentile. These findings suggest a strong correlation between HAZ-adjusted and unadjusted Z-scores given that both scores measure the same parameter. However, the reduced variability in the HAZ-adjusted Z-scores, particularly at the diagnostic threshold, is an important advantage of using adjusted scores because it reduces both underdiagnosis or overdiagnosis.

These findings are consistent with previous studies supporting size correction in children with growth disturbances, and they also reinforce the utility of methods such as bone volume estimation using geometric modeling approximations, as described by Kröger et al. (10). Nonetheless, these techniques also rely on certain assumptions and may not be routinely available in all clinical settings. Moreover, while newer imaging modalities like REMS may offer size-independent assessment (11), they are still not routinely available and further validation is required before widespread implementation in pediatric care.

Given these results, we believe that densitometric size adjustment should be applied to the whole pediatric population, regardless of stature or growth status. Size adjustment could be particularly beneficial in individuals whose height falls in the upper percentiles as unadjusted scores in this group are more likely to appear within the normal range, thus masking an LBMD diagnosis and delaying appropriate follow-up or treatment. Therefore, we recommend that clinicians consider applying size adjustment for all pediatric densitometry in all patients.

### 4.1 Strengths and limitations

The main limitation this study is the single-center design, which may limit the generalizability of our findings to other clinical settings or populations. Notwithstanding that limitation, the study hospital is a tertiary care referral center for rheumatic diseases, with a broad catchment area (> 450,000 residents), thus ensuring a diverse patient population. Another limitation is technical in nature, related to the DXA software used at the time of the study, which prevented us from performing Z-score evaluations for the TBLH projections. As a result, we were unable to obtain standardized assessments for those measurements. To overcome this limitation, we included both raw BMD values and Z-scores, which improves transparency and allows for a more nuanced interpretation of bone health in this population. It should be noted that this limitation is common in routine clinical practice at many hospitals, where lumbar spine DXA scans are often the only assessment performed. Despite this limitation, we believe it is important to present our data to encourage other centers to perform whole-body assessments, as this can provide important information until new software becomes available for TBLH projections (12). Clearly, these limitations should be considered when interpreting our findings. More research is needed with the latest DXA technology and standardized protocols across various populations to validate our findings.

This study has several strengths. First, several previous studies have concluded that size adjustment for DXA measurements should be performed in specific populations with growth disturbances (6, 13, 14). To our knowledge, however, our study is the first to investigate the need to perform size adjustments for all pediatric DXAs and the first to compare diagnostic discrepancies between adjusted and unadjusted measurements. By evaluating the impact of HAZ adjustment of Z-scores in a diverse, unselected pediatric cohort, our study provides evidence to support the benefits of size adjustment in improving the accuracy of the LBMD diagnosis. The inclusion of both raw and adjusted Z-scores further enhances the robustness of our analysis and supports the application of these findings in clinical practice.

### 4.2 Conclusion

In this study, we found discrepancies between HAZ-adjusted and unadjusted Z-scores in terms of the diagnosis of LBMD. More specifically, we found that the diagnosis differed in 7% of patients according to whether the adjusted or unadjusted scores were used. In 5% of cases, the patients no longer met criteria for a diagnosis of LDMD after HAZ adjustment. In 2% of cases, patients who originally had normal Z-scores met LBMD criteria after adjustment. These findings highlight the critical importance of performing size adjustment in all pediatric DXA measures to avoid both underdiagnosis and overdiagnosis. These findings suggest that Z-scores should be size adjusted for in all pediatric cases, especially in children in the top height percentiles, to improve the accuracy and reliability of the LBMD diagnosis.

## Data Availability

The original contributions presented in the study are included in the article. Further inquiries can be directed to the corresponding author/s.

## References

[1] Lalayiannis AD Crabtree NJ Fewtrell M Biassoni L Milford DV Ferro CJ. Assessing bone mineralisation in children with chronic kidney disease: what clinical and research tools are available? Pediatr Nephrol (Berlin Germany). (2020) 35:937–57. doi: 10.1007/s00467-019-04271-1 PMC7184042 31240395

[2] Gordon CM Leonard MB Zemel BS. Pediatric Position Development Conference: executive summary and reflections. J Clin densitometry: Off J Int Soc Clin Densitometry. (2013) 17:219–24. doi: 10.1016/j.jocd.2014.01.007 24657108

[3] Shuhart CR Yeap SS Anderson PA Jankowski LG Lewiecki EM Morse LR. Executive summary of the 2019 ISCD position development conference on monitoring treatment, DXA cross-calibration and least significant change, spinal cord injury, peri-prosthetic and orthopedic bone health, transgender medicine, and pediatrics. J Clin densitometry: Off J Int Soc Clin Densitometry. (2019) 22:453–71. doi: 10.1016/j.jocd.2019.07.001 31400968

[4] Bachrach LK Sills IN. Clinical report-bone densitometry in children and adolescents. Pediatrics. (2011) 127:189–94. doi: 10.1542/peds.2010-2961 21187316

[5] Binkovitz LA Henwood MJ. Pediatric DXA: technique and interpretation. Pediatr radiology. (2007) 37:21–31. doi: 10.1007/s00247-006-0153-y PMC1764599 16715219

[6] Salem N Bakr A. Size-adjustment techniques of lumbar spine dual energy X-ray absorptiometry measurements in assessing bone mineralization in children on maintenance hemodialysis. J Pediatr Endocrinol metabolism: JPEM. (2021) 34:1291–302. doi: 10.1515/jpem-2021-0081 34273916

[7] Crabtree N Ward K. Bone densitometry: current status and future perspective. Endocrine Dev. (2015) 28:72–83. doi: 10.1159/000223689 26138836

[8] Zemel BS Leonard MB Kelly A Lappe JM Gilsanz V Oberfield S. Height adjustment in assessing dual energy x-ray absorptiometry measurements of bone mass and density in children. J Clin Endocrinol Metab. (2010) 95:1265–73. doi: 10.1210/jc.2009-2057 PMC2841534 20103654

[9] Crabtree NJ Hogler W Cooper MS Shaw NJ. Diagnostic evaluation of bone densitometric size adjustment techniques in children with and without low trauma fractures. Osteoporosis international: J established as result cooperation between Eur Foundation Osteoporosis Natl Osteoporosis Foundation USA. (2013) 24:2015–24. doi: 10.1007/s00198-012-2263-8 23361874

[10] Kröger H Vainio P Nieminen J Kotaniemi A. Comparison of different models for interpreting bone mineral density measurements using DXA and MRI technology. Bone. (1995) 17:157–9. doi: 10.1016/S8756-3282(95)00162-X 8554924

[11] Fuggle NR Reginster J-Y Al-Daghri N Bruyere O Burlet N Campusano C. Radiofrequency echographic multi spectrometry (REMS) in the diagnosis and management of osteoporosis: state of the art. Aging Clin Exp Res. (2024) 36:135. doi: 10.1007/s40520-024-02784-w 38904870 PMC11192661

[12] Zemel BS Shepherd JA Grant SFA Lappe JM Oberfield SE Mitchell JA. Reference ranges for body composition indices by dual energy X-ray absorptiometry from the Bone Mineral Density in Childhood Study Cohort. Am J Clin Nutr. (2023) 118:792–803. doi: 10.1016/j.ajcnut.2023.08.006 37598746 PMC10579045

[13] Kor D Bulut FD Kılavuz S Şeker Yılmaz B Köşeci B Kara E. Evaluation of bone health in patients with mucopolysaccharidosis. J Bone mineral Metab. (2022) 40:498–507. doi: 10.1007/s00774-021-01304-4 35066680

[14] Ali F Cole CR Hornung L Mouzaki M Wasserman H Kalkwarf HJ. Age-related trajectory of bone density in children with intestinal failure: A longitudinal retrospective cohort study. JPEN J parenteral enteral Nutr. (2023) 47:736–45. doi: 10.1002/jpen.v47.6 PMC10875680 37227158

